# Financial Toxicity in Neuromyelitis Optica Spectrum Disorder: An ABC‐X Mixed‐Methods Study

**DOI:** 10.1002/brb3.71364

**Published:** 2026-04-22

**Authors:** Bo Wang, Shuchuan Tian, Zhaoxia Wang, Guilian Wang, Li Li, Jie Li, Huaxing Meng

**Affiliations:** ^1^ Department of Neurology First Hospital of Shanxi Medical University Shanxi China; ^2^ College of Nursing Shanxi Medical University Shanxi China; ^3^ Emergency Department Second Hospital of Shanxi Medical University Shanxi China

**Keywords:** Fear of progression, financial toxicity, mixed methods, neuromyelitis optica spectrum disorders, self‐efficacy, social support

## Abstract

**Background:**

Neuromyelitis Optica Spectrum Disorder (NMOSD), a rare antoimmune condition, is characterized by a high relapse rate, necessitating long‐term maintenance therapy even during clinically stable periods. This prolonged treatment regimen imposes a significant financial burden on patients, which may be greater than that associated with many other chronic diseases. However, data on the impact of financial toxicity (FT) specifically in NMOSD populations remain limited. This study aims to identify factors influencing FT in NMOSD patients using the ABC‐X model as a conceptual framework.

**Methods:**

A mixed‐methods study was conducted in two phases. In the quantitative phase, 210 NMOSD patients were evaluated using four validated scales: (1) Comprehensive Score for FT based on the Patient‐Reported Outcomes Measurement Information System (COST‐PROM), (2) Fear of Progression Questionnaire‐Short form (FoP‐Q‐SF), (3) Social Support Rating Scale (SSRS), and (4) general self‐efficacy scale (GSES). Multivariate linear regression analysis was performed to identify the predictors of FT. In the qualitative phase, 15 NMOSD patients were recruited via purposive sampling and interviewed using a semi‐structured format. Interview data were analyzed according to Colaizzi's seven‐step phenomenological method.

**Results:**

Quantitative findings indicated that fear of disease progression and lower self‐efficacy were significantly associated with higher levels of FT (*p* < 0.05). Qualitative analysis revealed four thematic categories characterizing the FT experience: (1) direct economic burden stemming from core illness‐related stressors, (2) insufficient or suboptimal utilization of available resources, (3) biased appraisal of stressors and maladaptive coping strategies, and (4) emergence of a multidimensional familial crisis state. The convergent results from both methodological approaches reinforced the study conclusions while enriching the overall understanding of FT in the population.

**Conclusion:**

This study demonstrates substantial FT among NMOSD patients, influenced by clinical, psychological, and social factors. These findings highlight the importance of integrating FT assessment into routine clinical care. In the Chinese healthcare context, challenges including delayed diagnosis, restricted access to medication, and high costs contribute significantly to this burden. Policy‐level interventions should focus on improving disease awareness, expanding treatment accessibility, and reducing financial strain on affected individuals and families.

## Graphical abstract

This mixed‐methods study shows 96.2% of Chinese NMOSD patients have financial toxicity from high biologic costs and job disruption; ABC‐X model analysis links fear of progression and low self‐efficacy to worse burden, multidimensional family crises and urgent policy and psychosocial support needs.

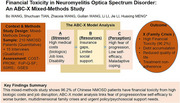

**Abbreviations**
FT: fnancial toxicity
NMOSD: neuromyelitis optica spectrum disorders

## 1 Introduction

Neuromyelitis optica spectrum disorders (NMOSD) are a group of autoimmune‐mediated inflammatory demyelinating diseases of the central nervous system (CNS), predominantly characterized by optic neuritis and transverse myelitis (Wingerchuk et al. 2015). The global prevalence of NMOSD ranges from 0.34 to 10 cases per 100,000 individuals, with higher rates reported in Asian populations (1.57–4.9 cases per 100,000) (Papp et al. 2021). In China, the incidence of NMSOD is estimated at 0.278–0.41 per 100,000 person‐years, reflecting a relatively large affected population (Y. Wu et al. 2022; Tian et al. 2020). Among patients with NMOSD, more than 90% experience recurrent relapsing‐remitting episodes. Without treatment, about 50% of patients develop severe visual or motor disability within 5–10 years (Kümpfel et al. 2023; Huang and Wu 2021). NMOSD frequently presents early with core clinical events defined by the 2015 diagnostic criteria (Wingerchuk et al. 2015), including optic neuritis (which may cause blindness), acute myelitis (which may cause paraplegia), area postrema syndrome, brainstem syndrome, diencephalic syndrome, and cerebral syndrome. Additional symptoms such as pain, anxiety, and depression are also common. With each relapse, neurological function progressively deteriorates. These symptoms and resultant disabilities impose substantial physical, emotional, and financial burdens on patients and their families, and significantly impair health‐related quality of life (Levy et al. 2022).

Financial toxicity (FT) refers to the adverse effects of medical treatment costs on patients and their families, encompassing both objective economic burden and subjective financial hardship (Y. Y. Zhang et al. 2023). This concept is currently applied in the context of chronic conditions such as cancer, as well as cardiovascular and cerebrovascular diseases (Elizondo et al. 2020). Patients' perceptions of their illness and their capacity to obtain effective support from external social resources have been shown to influence their experience of FT (Y. Y. Zhang et al. 2023; Yuan et al. 2022). In the field of neuroimmunology, multiple studies have documented a high prevalence of FT among patients with multiple sclerosis (MS), which is strongly associated with disability and costly treatment (Sadigh et al. 2021; Sadigh et al. 2022). However, in NMOSD—a condition characterized by a comparable disease burden and high treatment expenses—research on FT has significantly lagged.

A recent study of US patients with NMOSD provided the first systematic assessment of economic toxicity in this population. It revealed that over 75% of patients experience a significant economic burden, which is significantly associated with disability, race, and low income (Hill et al. 2025). These findings suggest that economic toxicity may represent a common but underrecognized core challenge in the management of NMOSD. However, differences in healthcare systems, levels of economic development, and sociocultural contexts may substantially influence patients' experiences of economic burden and their associated risk factors. Currently, there remains a paucity of studies on economic toxicity based on patient‐reported outcomes among NMOSD populations in China and across Asia.

The lack of effective therapies and the substantial financial burden are considered the main barriers to treatment access for patients with NMOSD (Illness Challenge Foundation 2020). FT may lead to psychological distress, depression, anxiety, and impaired quality of life (Tran et al. 2022; Banerjee et al. 2024). To cope with FT, patients may reduce spending on leisure activities, limit expenditures on basic necessities such as food and clothing, or deplete personal savings. More concerning, evidence suggests that patients may also exhibit reduced medication adherence and miss medical appointments—strategies aimed at minimizing costs—that could consequently precipitate disease relapse or clinical worsening (Ayub and Khan 2025).

The “ABC‐X” model, proposed by Reuben Hill in 1949, is a comprehensive theoretical framework for understanding family stress. It also serves as a foundational application of systems theory in analyzing and managing familial stress responses (Hobbs 1950). The model posits that stress does not originate from the event per se but emerges when an individual or family lacks sufficient functioning or resources to cope with a stressor (Schock‐Giordano 2013). The greater the negative impact of the stressor and the weaker the family's coping capacity, the more severe the resulting crisis. The model comprises four core components: “A” (Stressor) refers to stressor events—internal or external stimuli that elicit stress response within an individual or family system, encompassing both normative and non‐normative events; “B” (Resources) denotes the resources available for coping, including individual resources (knowledge, skills, and coping abilities, etc.), family resources (family cohesion, resilience, communication patterns, and functional capacity, etc.), and social resources (support from individuals, groups, and organizations outside the family). The availability and utilization of these resources influence how family members respond to stress; “C” (Family Definition) represents the cognitive appraisal, interpretation, and evaluation of the stressor by the individual or family—the perceived meaning and significance of the event—and reflects the extent to which available resources are mobilized, serving as a key determinant of stress severity; “X” (Family Crisis) signifies the outcome, specifically the degree of disruption or adverse impact the stressor exerts on the family system. Hill emphasized that a crisis (X) arises when a family perceives an event (A) as threatening (C) and believes that available resources (B) are inadequate to manage the challenge.

Based on the ABC‐X model and informed by recent US findings that 75% of NMOSD patients experience FT (COST < 25), with greater disability, higher attack frequency, and non‐White race associated with worse FT (Hill et al. 2025), we proposed the following quantitative hypotheses: (H1) clinical and socioeconomic stressors—including low household income, employment disruption, and greater disability—are positively associated with FT severity; (H2) fear of disease progression is positively associated with FT, while self‐efficacy is negatively associated with FT; and (H3) social support shows no significant association with FT. To complement these hypotheses, the qualitative component explored patients' experiences of financial stressors, barriers to resource access, and the role of illness perceptions in shaping coping strategies.

## 2 Materials and Methods

### 2.1 Ethics Approval

This study was approved by the Ethics Committee of the First Hospital of Shanxi Medical University (KYLL‐2024‐279). All participants provided written informed consent prior to enrollment and voluntarily agreed to participate in the study after receiving comprehensive information.

### 2.2 Study Design and Sample

We employed a mixed‐methods approach integrating quantitative and qualitative methodologies.

For the quantitative phase, sample size was estimated based on a rule of thumb of 5–10 times the number of independent variables (Ni et al. 2010). With an estimated 14 independent variables in the main analysis, the required sample size was calculated to be 70–140. After inflating this estimate by 20% to account for potential non‐response or invalid questionnaires, the target sample size was set at 88–175.

Consecutive inpatients diagnosed with NMOSD according to the 2015 international consensus criteria (Wingerchuk et al. 2015) were recruited from the Department of Neurology at the First Hospital of Shanxi Medical University between September 2024 and December 2024 for convenience‐sampling surveys. During this period, a total of 220 eligible patients were enrolled, exceeding the target sample size. The majority of these patients were admitted for scheduled disease‑modifying therapy (DMT) infusions or routine evaluations, with only a minority hospitalized due to acute exacerbations. Because most admissions were for maintenance therapy rather than acute episodes, we were able to recruit a large, clinically stable cohort efficiently. Recruitment was terminated at the end of the planned enrollment window.

We provided standardized clarifications for the questionnaire. Using quantitative findings, theoretical frameworks, and existing literature, we developed an interview protocol and conducted qualitative interviews from December 2024, to January 2025. Through purposive sampling, NMOSD patients who participated in the initial quantitative phase were selected for semi‐structured interviews. In the initial qualitative component of the study, both semi‐structured interviewing techniques and phenomenological research methods were employed.

The enrollment criteria are as follows: (1) age ≥18 years, (2) NMOSD was diagnosed according to the 2015 international consensus criteria for NMOSD (Wingerchuk et al. 2015), and (3) understanding of the study objectives and procedures. The exclusion criteria are: (1) Congenital limb dysfunction; (2) Inability to provide informed consent or cooperate with assessments due to concomitant psychiatric disorders.

### 2.3 Measurement Indicators for Quantitative Research

The data collection form for general demographic and disease‐related characteristics includes age, gender, disease duration, educational level, occupation, residential location, medical expense payment forms, methods of medical expense payment, number of disease episodes, number of concomitant chronic conditions, and the Expanded Disability Status Scale (EDSS) score.

The Comprehensive Score for FT based on Patient‐Reported Outcome Measures (COST‐PROM) was employed in this study. Originally developed by Souza et al. at the University of Chicago in 2014, this tool was later translated into Chinese by domestic researchers in 2017 (De Souza et al. 2015; Yu et al. 2017). The scale consists of 11 items grouped into three domains: financial expenditure, financial resources, and psychosocial responses. A 5‐point Likert scale is used (0 = not at all; 4 = very much), yielding a total score ranging from 0 to 44. A score of ≤ 25 indicates the presence of FT, with lower scores reflecting more severe FT. Scores ≤ 17.5 were classified as moderate‐to‐severe FT, representing clinically significant burden (Ng et al. 2021). The Chinese version of the scale demonstrated good reliability and validity, with a Cronbach's α coefficient of 0.893.

The Chinese version of the Fear of Progression Questionnaire‐Short Form (FoP‐Q‐SF) was translated by Wu et al. This instrument comprises two domains: physiological well‐being (six items) and social/family aspects (six items). Each of the 12 items is rated on a 5‐point Likert scale (1 = never; 5 = always), yielding a total score ranging from 12 to 60. Higher scores indicate a more severe fear of disease recurrence. A total score of 34 or higher suggests a clinically significant level of fear regarding disease progression. Due to its strong reliability and validity, this scale has been widely utilized (Hinz et al. 2015). The scale demonstrated a Cronbach's alpha coefficient of 0.883, confirming its good internal consistency and psychometric robustness (Q. Wu et al. 2015).

Participants' levels of social support were assessed using the Social Support Rating Scale (SSRS) developed by Xiao Shuiyuan (Xiao 1994). The scale consists of 10 items that evaluate three domains: objective support, subjective support, and utilization of support. Items 1–5 and 8–10 are rated on a 4‐point Likert scale (1 = “not at all,” 4 = “very much”). Items six and seven are scored as follows: “no source” = 0, and each listed source under “have a source” = 1. Total scores range from 12 to 66, with higher scores indicating greater perceived social support. In this study, the scale demonstrated good internal consistency (Cronbach's *α* = 0.83).

The Chinese version of the general self‐efficacy scale (GSES), adapted by Wang Caikang and colleagues in 2001 from the original scale developed by Schwarzer and Jerusalem (Schwarzer and Jerusalem 1995), was utilized. Translated into 30 languages, the GSES is widely used in health research. This 10‐item instrument employs a 5‐point Likert scale (ranging from 0 to 4) to assess individuals' general perceived self‐efficacy. Total scores, calculated by summing item responses, range from 0 to 40, with higher scores indicating greater levels of self‐efficacy. The scale demonstrated excellent internal consistency (Cronbach's *α* = 0.960) (C. K. Wang et al. 2001, Y. L. Wang et al. 2020).

### 2.4 Interview Guide for Qualitative Investigation

The interview framework was developed based on findings from prior quantitative studies, a comprehensive review of the relevant literature, and input obtained through expert consultations. The protocol was refined iteratively during the interview process, incorporating insights gained from two pilot interviews with patients. A detailed description of each component is provided in Additional file 1 online.

### 2.5 Data Collection and Quality Control Methods

During the quantitative phase, standardized protocols were strictly followed to ensure explanation of the study purpose, significance, safety measures, and data accuracy and objectivity. Questionnaires were collected by researchers prior to patient discharge. Prior to each interview, the participant and researcher mutually agreed upon the location, time, and format of the interview. Semi‐structured interviews were conducted during the qualitative phase to explore patients’ experiences with FT.

Participants were recruited from individuals who completed the questionnaire and consented to participate in interviews. To ensure maximum variation and capture the diversity of NMOSD patient experiences, we stratified the sampling frame according to the following key characteristics: gender, age group, educational level, occupation, method of medical expenses payment, EDSS, and medication regimen. Within each stratum, participants were purposively selected and invited for interviews until data saturation was achieved (Morse 1991; Polit and Beck 2008), meaning that subsequent interviews no longer yielded new themes or insights. All interviews were semi‐structured, one‐on‐one, and audio‐recorded with permission. Researchers monitored participants' emotional responses and facial expressions throughout the interviews. Interview transcripts were cross‐verified by two researchers to ensure accuracy. Each interview lasted approximately 30 min and was conducted in the nurse manager's office to ensure privacy and comfort.

### 2.6 Data Analysis

#### 2.6.1 Quantitative Data

Quantitative data were analyzed using SPSS 26.0. The COST‐PROM consists of 11 items rated on a 5‐point Likert scale (0–4), with each item representing an ordinal level of measurement. In the original development and validation of the COST measure, the authors explicitly considered the item scores to be ordinal (De Souza et al. 2015). Following this methodological precedent, we treated COST‐PROM scores as ordinal data. We employed non‐parametric tests for all analyses involving FT to ensure statistical rigor and avoid assumptions about interval‐level properties.

Non‐normally distributed measurement data were expressed as median (interquartile range), and Spearman correlation analysis was performed to assess associations. Categorical data were presented as frequency and percentage, and intergroup comparisons were conducted using the *χ* ^2^ test. For patients with NMOSD, non‐parametric tests were used for univariate analysis in comparison with FT. The Mann‐Whitney *U* test was applied for two‐group comparisons, and the Kruskal–Wallis *H* test was used for multiple‐group comparisons. Significant univariate predictors (*p* < 0.05) were included in a multivariate linear regression model to identify factors associated with FT in NMOSD patients.

#### 2.6.2 Qualitative Data

For the qualitative data, we iteratively reviewed the entire transcript and conducted a line‐by‐line thematic analysis (Braun and Clarke 2006; Elo and Kyngäs 2008; Flick 2018).

Two expert qualitative researchers performed the analysis using NVivo 15 software. Methodological rigor was anchored in Guba and Lincoln's framework (Guba and Lincoln 1981), encompassing confirmability, dependability, transferability, credibility, and fittingness (Carnevale 2002).

Two researchers independently coded the interview transcripts to ensure the study's credibility and transferability. Auditability was established by systematically documenting data‐related decisions in field notes, memos, and reflexivity journals. Fittingness was strengthened through iterative researcher checks to confirm that interpretations were consistent with participants’ lived experiences.

#### 2.6.3 Methodological Rigor in Connection With Mixed Methods Research

Accorfing to Fetters et al. (2013), joint displays were employed to visually integrate mixed‐methods data, thereby generating insights that transcend those derived from standalone analyses.

Guided by existing literature (Younas et al. 2019; Younas et al. 2020; Guetterman et al. 2015), we developed a synthesis table linking qualitative themes with quantitative findings to examine areas of convergence and divergence across the two datasets.

To ensure methodological rigor, we applied legitimation criteria informed by methods research principles (Younas et al. 2020; Onwuegbuzie and Johnson 2006).

## 3 Results

### 3.1 Results of a Quantitative Study

#### 3.1.1 General Information on the Study Population

In this study, 220 questionnaires were distributed. After excluding 10 questionnaires with incomplete data, 210 valid responses were obtained, yielding an effective response rate of 95.5%. Among the 210 patients (median age 43.5 years [interquartile range: 32–54]; 13.8% male), educational attainment levels were as follows: ≤ primary school, 20% (*n* = 42); junior high, 24.3% (*n* = 51); high school or technical secondary school, 16.7% (*n* = 35); college or bachelor's degree, 36.7%; and master's degree and above, 2.4% (*n* = 5) (Table 1).

**TABLE 1:** Results of univariate analysis on the factors of financial toxicity among NMOSD patients (*n* = 210).

Characteristics		*N* (%)	FT	*r/Z*	*p*
Education level^a^				12.153	0.016
	A primary school and below	42 (20)	8.50 (2.50, 14.00)		
	Junior high school	51 (24.3)	11 (5.00, 15.00)		
	High school or technical secondary school	35 (16.7)	10 (4.00, 14.00)		
	College or Bachelor's Degree	77 (36.7)	11 (6.50, 19.50)		
	Master's degree and above	5 (2.4)	16.00 (14.50, 24)		
Average monthly household income (CNY)^a^				18.602	< 0.001
	< 1500	56 (26.7)	9.00 (4.00, 12.00)		
	1500–4999	109(51.9)	10.00 (3.00, 17.00)		
	>5000	45 (21.4)	15.00 (10.00, 22.50)		
Comorbidities^a^				14.41	< 0.001
	No comorbidities	43 (20.5)	16.00 (10.00, 22.00)		
	One comorbidity	83 (39.5)	10.00 (3.00, 16.00)		
	More than one comorbidity	84 (40.0)	9.00 (4.00, 14.00)		
Employment at diagnosis^a^				21.829	< 0.001
	Government, SOE, or public sector	41 (19.5)	15.00 (7.00, 20.00)		
	Private sector or self‐employed	36 (17.1)	14.00 (9.25, 23.00)		
	Agriculture	59 (28.1)	12.00 (5.00, 15.00)		
	Unemployed or doing housework	74 (35.2)	9.00 (2.75, 12.00)		
EDSS^a^				24.288	< 0.001
	Mild neurological impairment	75 (35.7)	14.00 (8.00, 22.00)		
	Moderate neurological impairment	36 (17.1)	13.00 (7.00, 19.00)		
	Severe neurological impairment	99 (47.2)	9.00 (3.00, 12.00)		
Change of employment status (conditional on employed at diagnosis) ^a^				18.427	< 0.001
	No change	76 (36.2)	14.00 (8.25, 21.75)		
	Completely stop working	105 (50.0)	10.00 (4.00, 23.00)		
	Change positions	29 (13.8)	9.00 (4.00, 18.50)		
FoP‐Q‐SF^b^		42 (37,51)		−0.629	< 0.001
	Physiological well‐being	21 (18,24)		−0.566	< 0.001
	Social/family	21 (18,27)		−0.614	< 0.001
GSES^c^		19 (15,22)		0.445	< 0.001
SSRS^a^		34 (28,39)		0.175	0.011
	Objective support	7 (6,8)		0.236	< 0.001
	Subjective support	21 (15,25)		0.113	0.103
	Utilization of support	6 (5,8)		0.103	0.136

*Note*: Data are expressed as counts and medians (25th–75th percentiles). This table shows only statistically significant data. Calculated based on the average exchange rate for 2025: $1≈7.15 CNY; <¥1500,≈<$210; ¥1500–4999,≈$210–699; >¥5000,≈>$699.

Abbreviation: FT, financial toxicity.

^a^ Analyzed with Kruskal–Wallis *H* test (Z).

^b^ Analyzed via Mann–Whitney *U* test (Z).

^c^ Analyzed using Spearman correlation analysis (*r*).

#### 3.1.2 FT Score of NMOSD Patients

The FT among NMOSD patients was assessed using the COST‐PROM instrument. Descriptively, the mean COST‐PROM score was 11.19 (SD = 7.86), which was nearly identical to the median score of 11.00 (IQR: 4.00, 17.00). The distribution exhibited mild positive skewness (0.502) and near‐normal kurtosis (0.025). Formal Shapiro–Wilk testing (*W* = 0.957, *p* < 0.001) indicated a slight deviation from a strictly normal distribution. The prevalence of any degree of FT (COST‐PROM ≤ 25) was 96.2%. Using the validated cut‐off score of 17.5 to indicate high FT (Ng et al. 2021), 80.5% of patients were identified as experiencing moderate‐to‐severe FT. A detailed breakdown of the score distribution is provided in Table 2.

**TABLE 2:** Distribution details of financial toxicity (COST‐PROM) scores among NMOSD patients (*N* = 210).

Item	Value
(COST‐PROM > 25). *n* (%)	8 (3.8%)
(COST‐PROM ≤ 25), *n* (%)	202 (96.2%)
**Descriptive statistics**	
Mean (SD)	11.19 (7.86)
Median (IQR)	11.00 (4.00, 17.00)
Min, Max	0,00, 42.00
Skewness/Kurtosis	0.502/0.025

**FIGURE 1:**
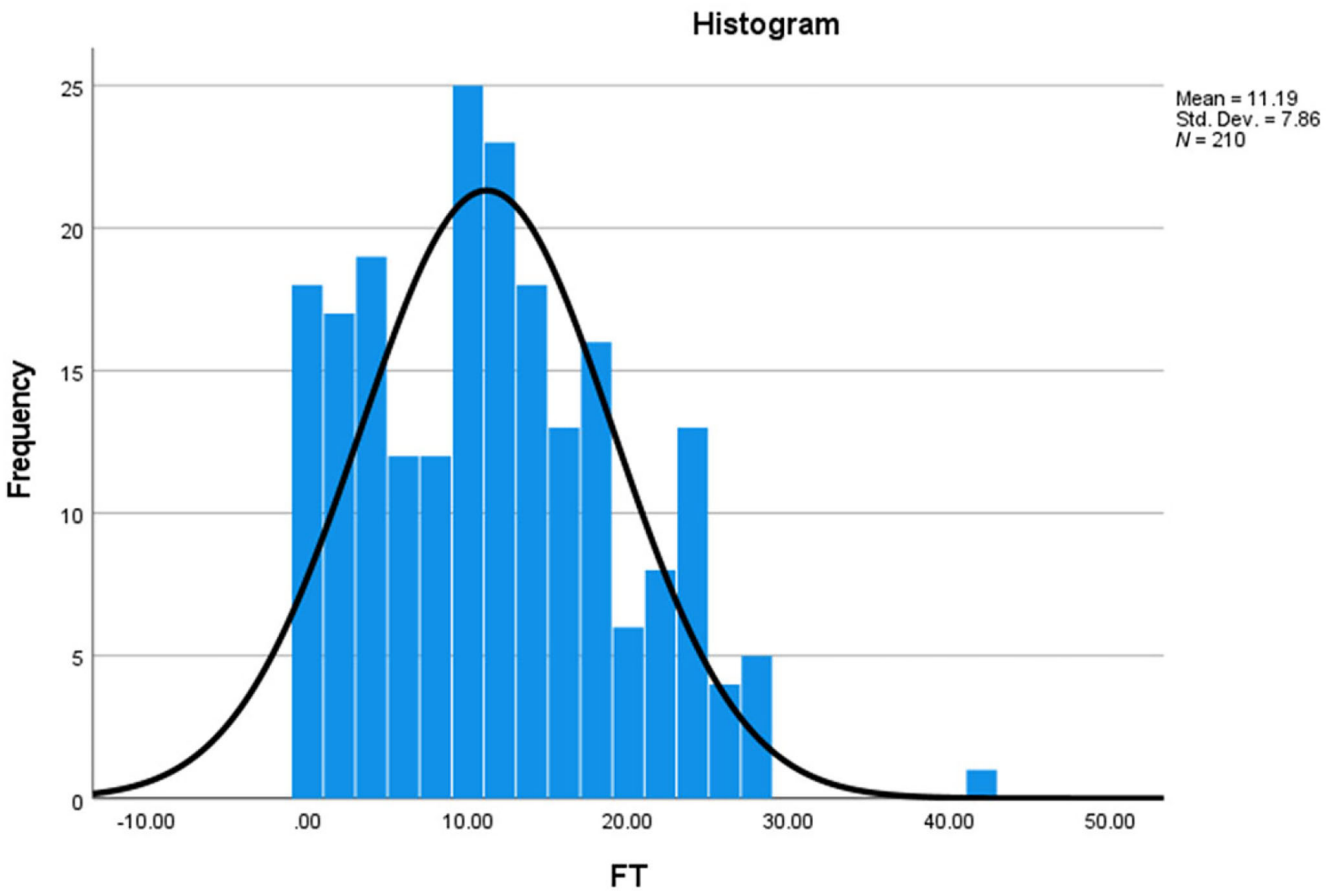
Distribution of Financial Toxicity (COST‐PROM) Scores among NMOSD Patients (*N* = 210).

Visual inspection of the histogram (Figure 1) confirms a predominantly bell‐shaped distribution with a mild right tail, which is driven by a small subset of outliers (3.8% of patients with COST‐PROM > 25). We used non‐parametric tests for univariate analyses as a conservative statistical practice, given the approximately normal distribution with mild tail asymmetry. More importantly, following the original developers' approach, we treated COST‐PROM scores as fundamentally ordinal data and applied non‐parametric methods accordingly, as they used Spearman correlations in validation based on the Likert‐scale item structure (De Souza et al. 2015).

#### 3.1.3 Univariate Analysis on Features Associated With FT in NMOSD Patients

Univariate analysis identified multiple factors significantly associated with FT (COST‐PROM score). Results are summarized below, with detailed data presented in Table 1.

Demographic and clinical factors: FT was significantly more severe among patients with lower monthly household income (*z* = 18.602, *p* < 0.001), higher disability levels (EDSS score) (*z* = 24.288, *p* < 0.001), and presence of comorbidities (*z* = 14.41, *p* < 0.001). Patients who were unemployed at diagnosis (*z* = 18.427, *p* < 0.001) or experienced a negative change in employment status after diagnosis (*z* = 18.427, *p* < 0.001) also exhibited significantly higher levels of FT. Significant differences existed in employment at diagnosis (*z* = 21.826, *p* < 0.001), with patients who were “unemployed or doing housework” bearing the heaviest FT. Second, among patients employed at diagnosis, post‐diagnosis employment changes also exhibited a clear gradient relationship (*z* = 18.427, *p* < 0.001): patients who “changed jobs” experienced the most severe FT, followed by those who “completely stopped working,” while those with “no change in employment” bore the lightest burden. Additionally, education level (*z* = 12.153, *p* = 0.016) was also significantly correlated with FT, showing a trend where lower educational attainment was associated with higher FT scores.

Psychological and Social Factors: Fear of disease progression (FoP‐Q‐SF) showed a strong negative correlation with FT (r = −0.629, *p* < 0.001), indicating that stronger fear was associated with greater perceived FT. Conversely, GSES (*r* = 0.445, *p* < 0.001) and Social Support (SSRS) (*r* = 0.175, *p* = 0.011) were positively correlated with FT, indicating that higher self‐efficacy and stronger social support are associated with lower perceived financial burdens.

#### 3.1.4 Multiple Linear Regression Modeling of Factors Associated With FT in NMOSD Patients

Multiple linear regression analysis identified six factors independently associated with FT. The model explained 51.5% of the variance in the total FT score (*R* ^2^ = 0.515) and exhibited residual independence (Durbin–Watson = 2.026). The final model identified six independent predictors (Table 3).

**TABLE 3:** Multiple linear regression modeling of factors associated with FT in NMOSD patients.

Characteristics	B (SE)	*β*	*t*	*p* value	(95% CI)	Model summary
Average monthly household income	2.310 (0.629)	0.204	3.676	< 0.001	(1.071,3.550)	*F* = 19.136 *p* < 0.001 Adjusted *R* ^2^ = 0.488
Change of employment status	−1.408 (0.617)	−0.120	−2.282	0.024	(−2.626,−0.191)	*R* ^2^ = 0.515
Employment	−0.994 (0.417)	−0.142	−2.381	0.018	(−1.817,−0.171)	
Physiological well‐being	−0.352 (0.146)	−0.213	−2.403	0.017	(−0.641,−0.063)	
Social/family	−0.380 (0.130)	−0.274	−2.925	0.004	(−0.636, −0.124)	
GSES	0.209 (0.088)	0.153	2.382	0.018	(0.036,0.381)	

To interpret the real‐world magnitude of effects, we report the unstandardized regression coefficients (*B*), which represent the average change in the FT score (in points) associated with a one‐unit increase in the predictor (or for categorical variables, compared to the reference group). The standardized coefficients (β) are also provided for comparing the relative strength of associations among variables.

Household Economic Status: A lower average monthly household income was strongly associated with greater FT. For each decrease in income category (e.g., from the high‐income category [>¥5000,≈>$699] to the middle‐income category [¥1500–4999,≈$210–699]), the FT score decreased by an average of 2.31 points (*B* = 2.310, 95% CI: 1.071–3.550; *p* < 0.001), indicating a significant worsening of FT.

Employment Impact: Negative change in employment status after diagnosis was independently associated with worse FT. Compared to patients with no change, those who experienced a negative employment change had FT scores that were an average of 1.41 points lower (*B* = ‐1.408, 95% CI: −2.626 to −0.191; *p* = 0.024). Furthermore, being unemployed at diagnosis was associated with a 0.99‐point lower score compared to being employed (*B* = −0.994, 95% CI: −1.817 to −0.171; *p* = 0.018).

Psychosocial Factors: Better psychosocial resources were protective. Each 1‐point increase in the GSES score was associated with a 0.21‐point increase in the FT score (*B* = 0.209, 95% CI: 0.036–0.381; *p* = 0.018). Similarly, higher social/family well‐being (*B* = 0.380, 95% CI: 0.124–0.636; *p* = 0.004) and better physiological well‐being (*B* = 0.352, 95% CI: 0.063–0.641; *p* = 0.017) were associated with significantly higher FT scores (i.e., lower toxicity).

**FIGURE 2:**
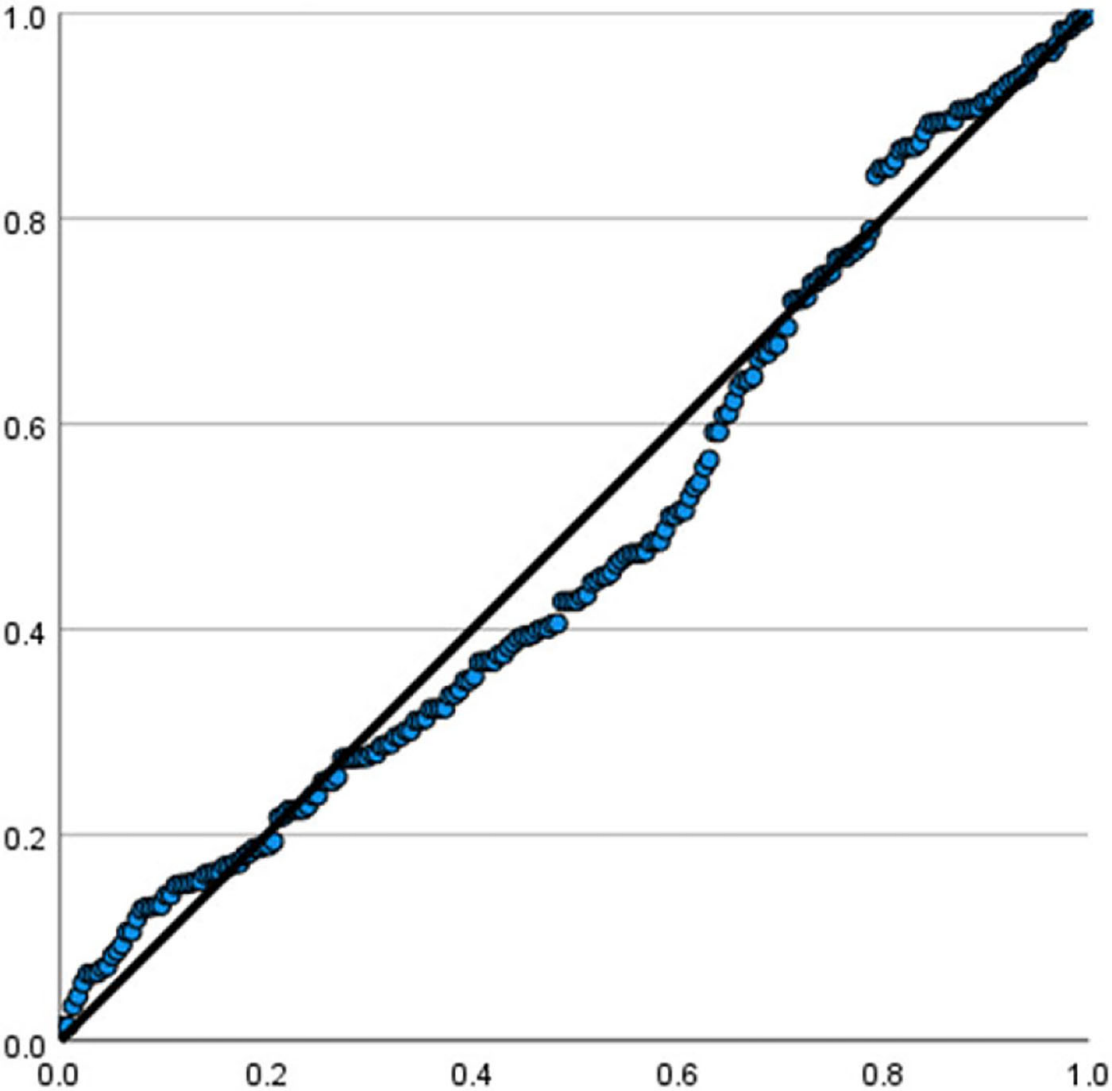
Normal P‐P Plot of the Standardized Residuals from the Multivariable Linear Regression Model.

**FIGURE 3:**
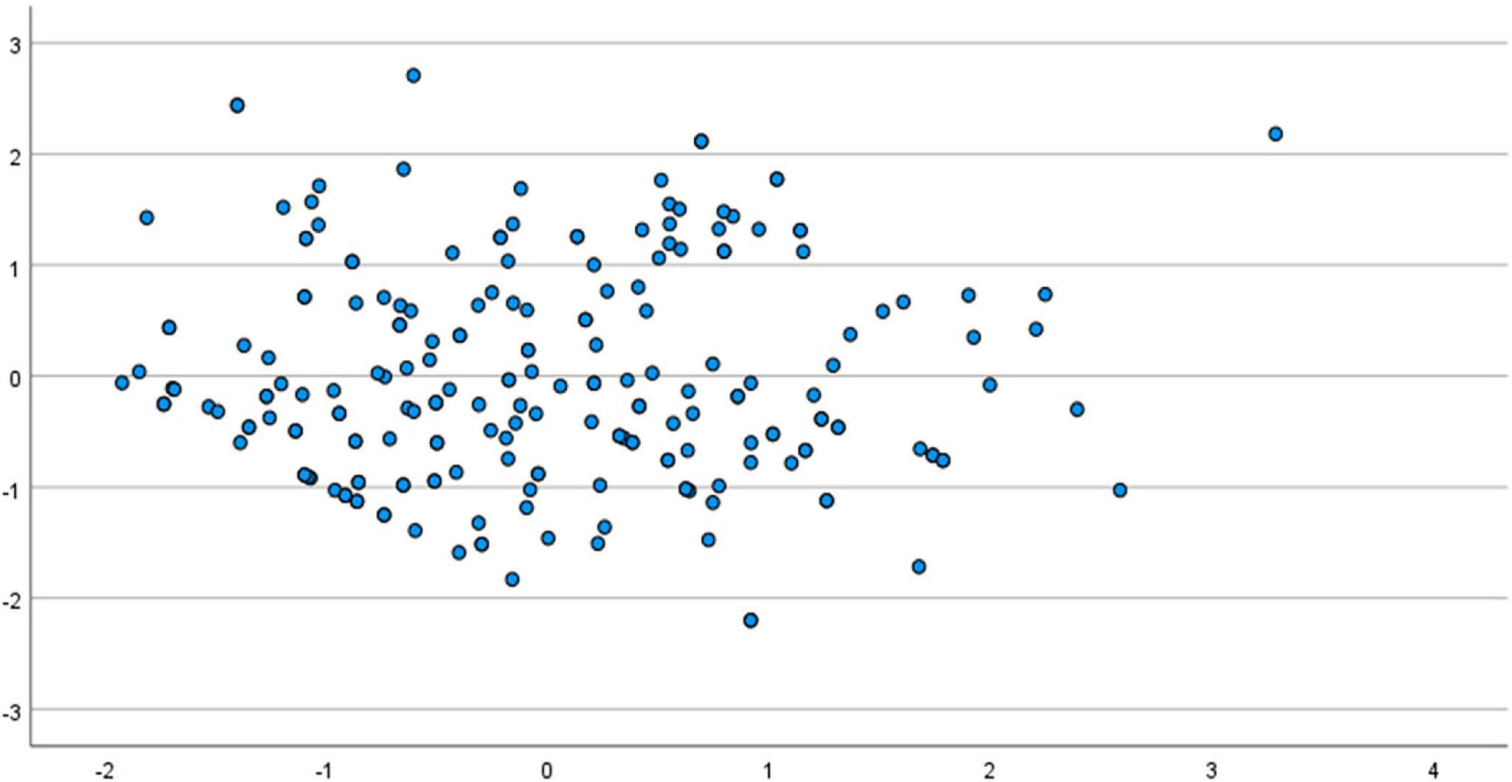
Scatterplot of Standardized Residuals versus Standardized Predicted Values from the Multivariable Linear Regression Model.

Diagnostic checks for the linear regression model were conducted. A normal P‐P plot of the standardized residuals and a scatterplot of residuals versus predicted values (Figures 2 and 3) showed no severe violations of the normality and homoscedasticity assumptions, supporting the robustness of the model.

### 3.2 Outcomes of Qualitative Study

#### 3.2.1 Features of the Enrolled Patients

In this qualitative component, sampling continued until thematic saturation was reached, following the stratified purposive sampling strategy detailed in Section 2.5. A total of 15 NMOSD patients (10 females, 5 males; see Table 4) were interviewed, at which point subsequent analysis confirmed that no new themes were emerging.

**TABLE 4:** Patients' general information (*n* = 15).

Number	Gender	Age	Methods of medical expenses payment	Medication	Education level	Occupation	EDSS	Comorbidities	Time since diagnosis(years)
P1	Female	50	Self‐pay	Rituximab	Secondary school	Unemployed	Severe	1	7
P2	Female	20	Self‐pay	Rituximab	University education	Freelancer	Mild	0	1
P3	Female	45	Medical insurance	Inebilizumab	Secondary school	Farmer	Severe	2	15
P4	Female	60	Self‐pay	Hormones	Primary school	Farmer	Moderate	1	5
P5	Female	64	Self‐pay	Inebilizumab	Primary school	Freelancer	Moderate	1	5
P6	Female	51	Medical insurance	Rituximab	Secondary school	Employee	Mild	2	10
P7	Female	53	Self‐pay	Rituximab	Primary school	Farmer	Severe	3	20
P8	Female	50	Medical insurance	Rituximab	University education	Worker	Mild	0	3
P9	Female	64	Self‐pay	Rituximab	Primary school	Farmer	Severe	2	12
P10	Female	54	Medical insurance	Inebilizumab	Secondary school	Worker	Mild	1	2
P11	Male	41	Self‐pay	Rituximab	University education	unemployed	Severe	3	6
P12	Male	25	Medical insurance	Inebilizumab	University education	Employee	Mild	0	2
P13	Male	42	Medical insurance	Inebilizumab	University education	Worker	Moderate	0	5
P14	Male	56	Self‐pay	Rituximab	Secondary school	Freelancer	Severe	2	21
P15	Male	55	Self‐pay	Rituximab	Secondary school	Farmer	Moderate	2	11

#### 3.2.2 Topics of this Qualitative Investigation

In this study, interview data from 15 patients with NMOSD were thoroughly analyzed, compared, and summarized, resulting in four major themes and 12 minor themes.

##### 3.2.2.1 Theme I: Core Stressful Events can Lead Directly to Increased Economic Burdens

###### 3.2.2.1.1 High Direct Medical Costs

The main ones include the convoluted diagnostic process, the out‐of‐pocket pressure of high‐priced biologics, and the cost of side effects of alternative programs. Most patients go through a long and tortuous process of seeking medical treatment before being diagnosed with NMOSD, which not only consumes a lot of time and money but also causes both physical and psychological torture; patients' annual treatment cost generally exceeds their family's annual income, and they rely on borrowing or selling their assets, and low‐cost alternative treatments lead to osteoporosis, infections, and other complications, which further pushes up the healthcare expenditures. “A shot of Inebilizumab costs over 20,000 RMB (≈$2797 USD) out of pocket, and my family's savings are gone.” (P5); “After 20 years of treatment, the family's savings have been used up and we've sold all our wheat.” (P7); “Hormones save money on meds, but necrotic femur surgery costs ¥80,000 (≈$11189 USD).” (P4); “At the beginning, because of the numbness of the right thumb and eye problems, I was treated in the ophthalmology department of the county hospital, and then the effect was not good, so I went to Beijing to see a doctor to confirm the diagnosis. It took seven to eight years from the first discomfort to the diagnosis.” (P3); “Initially misdiagnosed, stayed in neurosurgery, should have been around 15 hospitalizations in 4 years, cost over ¥100,000 (≈$13986 USD) after reimbursement, only diagnosed with optic neuromyelitis optica 6 months ago.” (P4)

###### 3.2.2.1.2 Unsustainability of Long‐Term Treatment

Families of patients who have had the disease for more than 10 years are caught in a vicious cycle of “treatment‐debt‐relapse”. “After 10 years of treatment, now I can't even borrow money.” (P6)

###### 3.2.2.1.3 Disease Disability and Career Breaks

Loss of sight or physical disability forces patients to leave their jobs, and the family is dependent on a single member for income. “Blind in my right eye and only 0.3 in my left eye, which organization dares to take me?” (P1)

##### 3.2.2.2 Theme II: Insufficient or Inefficient use of Resources Available to Households

###### 3.2.2.2.1 Limitations of Policy Support

High thresholds for major medical assistance, reimbursement limitations for off‐site visits, and non‐inclusion of high‐priced drugs in medical insurance. Patients are unable to apply for Major Medical Assistance due to the number of hospitalizations or the amount of out‐of‐pocket expenses not being met. Biologics are not included in local health insurance reimbursement catalogs, and patients are forced to pay the full amount out‐of‐pocket. Low reimbursement rates for interprovincial treatment and the burden is exacerbated by transportation and accommodation costs. “You have to spend enough to get ¥50,000 (≈$6993 USD) for a hospitalization for major medical assistance, and I only spend ¥30,000 at a time, which is never enough.” (P9); “Chronic medical insurance can only cover some antihypertensive drugs, and I have to buy all my specialty drugs myself.” (P8); “When you go to Beijing to see a doctor, the new rural cooperative medical insurance only covers 30% of the cost, and the cost of traveling is more expensive than the cost of medicine.” (P3)

###### 3.2.2.2.2 Vulnerability of Social Networks

Difficulties in borrowing from friends and relatives and barriers to access to charitable assistance. Often patients with FT have relatives who may not be financially well off either and can offer little help. Patients give up applying for social assistance due to lack of information or stigma. “They're having a hard time. I don't want to borrow money from them.” (P12); “I didn't know there was charitable aid and I didn't know how to get it.” (P5); “When it comes to borrowing money, they don't look good.” (P13)

###### 3.2.2.2.3 Breakage of the Family Economic Chain

Families cut back on basic expenses such as education and food in order to pay for medical care. “The children dropped out of school to work, and the family hasn't bought new clothes in three years.” (P2)

##### 3.2.2.3 Theme III:Assessment of Stressors and Selection Bias in Coping Strategies

###### 3.2.2.1 Economic Compromise and Suboptimal Treatment Options

“Inebilizumab after the hormone flush, which costs $60,000 (≈$8392 USD) a year, can only be selected semi‐annually.” (P3)

###### 3.2.2.2 Lack of Awareness of Policy and Charitable Assistance

“I've never applied for Social Security Major Medical Assistance and I don't know what to do about it.” (P4); “I don't know how to apply for charity” (P7)

###### 3.2.2.3 Stigma and Help‐Seeking Avoidance

Patients give up applying for social assistance due to stigma. Hormone‐induced obesity or disability triggers low self‐esteem, and patients avoid social activities. “I didn't apply for charity because I was afraid my classmates would find out I was sick.” (P2); “I feel ashamed to apply for these charitable aid from Waterdrop Fundraising.” (P5); “I've gained 50 pounds, I wear a mask when I go out, and I don't want to see anyone.” (P12); “I am afraid to apply for medical reimbursement for major illnesses for fear that it will affect my ability to find a job in the future.” (P10)

##### 3.2.2.3 Theme IV: Multidimensional Family Crisis State Formation

###### 3.2.2.3.1 Economic Collapse and Debt Accumulation

Families cut back on basic expenses such as education and food in order to pay for medical care. “The children dropped out of school to work, and the family hasn't bought new clothes in three years.” (P9); “Now that I have 100,000 (≈$13986 USD) in bank loans, I can barely afford to eat.” (P14); “The family's savings are depleted, and relatives can't borrow any more.” (P6)

###### 3.2.2.3.2 Quality of Life Falls off a Cliff

“Children's clothes are saved, and my family hasn't bought meat in three years.” (P9); “Entertainment? No energy or money.” (P3)

###### 3.2.2.3.3 Impairment of Psychological and Social Functioning

“Wearing a mask when I go out, afraid that people will say that I always have accidents at home.” (P15); “Low self‐esteem to the point where I am afraid to meet people and can only lie down at home.” (P2)

### 3.3 Mixed Findings

The combined presentation integrating qualitative and quantitative results is presented in Table 5.

**TABLE 5:** Joint presentation of qualitative and quantitative data.

ABC‐X	Quantitative results	Qualitative results	Conclusion
X (family crisis)	COST‐PROM the financial toxicity score was 11.00 (4.00, 17.00)	Multidimensional family crisis state formation	The high incidence of financial toxicity was 96.2%. Qualitative studies have clarified that patients with financial toxicity are prone to family crisis states such as debt accumulation, reduced quality of life, and impaired psychological and social functioning. Qualitative studies confirm the manifestation of financial toxicity in patients.
A (stressor)	Household income; Employment at diagnosis; change of employment status	High direct medical costs, unsustainable long‐term treatment, disability, and career interruption all contribute directly To increased FT	Both quantitative and qualitative data show that it is an important issue for patients, which has a strong impact on quality of life.
B (resources)	The difference in the effect of social support on patients' financial toxicity was not statistically significant (*p* > 0.05).	Insufficient or inefficient use of resources available to households	Quantitative results show the relationship between social support and financial toxicity. The qualitative results further expand on the core issues of inadequate social support, including insufficient health insurance coverage, depletion of borrowing from family and friends, and barriers to charitable assistance acquisition
C (perception)	Fear of progression (physiological well‐being: *β* = −0.213, *p* = 0.017; Social/family: *β* = −0.274, *p* = 0.004) was positively predictive of FT. Self‐efficacy (*β* = 0.153, *p* < 0.018) was a negative predictor of FT.	Assessment of stressors and selection bias in coping strategies.	Quantitative results shed light on the relationship between fear of disease progression and self‐efficacy with FT. Qualitative results clarify the assessment of financial burden and selection of coping strategies bias in the treatment of patients with NMOSD

## 4 Discussion

This study reveals a substantial burden of FT among NMOSD patients in China, with 96.2% experiencing some degree of FT and 80.5% reporting moderate‐to‐severe levels. These figures indicate that the economic impact of NMOSD extends far beyond routine out‐of‐pocket expenses, encompassing significant psychological distress and material hardship.

The substantial FT burden observed in this NMOSD cohort invites comparison with other chronic conditions that share similar clinical and economic features. MS, as the most direct comparator, affects a similar demographic and requires long‐term disease‐modifying therapies. A US study of 243 MS patients using the same COST‐PROM measure reported a mean score of 17.4 ± 10.2 (Sadigh et al. 2021), indicating less toxicity than our cohort. Both studies identified similar risk factors—prior relapse history in MS mirrored our finding that greater disability predicts FT. Evidence from Japanese cancer patients offers insights from a comparable Asian healthcare context. A recent review found that while 70% of patients utilized financial support systems, awareness and actual utilization remained suboptimal (Itani et al. 2023), paralleling our qualitative finding that patients often fail to access available assistance. These cross‐disease and cross‐cultural comparisons underscore that while FT is a shared concern across serious illnesses, its magnitude is shaped by disease‐specific factors, healthcare system design, and the accessibility of support mechanisms.

This finding aligns with and extends the emerging international evidence on FT in NMOSD, while highlighting important contextual differences. A direct comparison with the recent study by Hill et al. (2025) reveals both shared burdens and instructive disparities. Both studies consistently identify low household income and greater disability as core, cross‐cultural determinants of FT. However, the prevalence of FT in our cohort is substantially higher. This difference likely arises from several methodological factors. First, our study had a larger sample size. Second, although both studies used convenience sampling, we employed a convergent mixed‐methods approach that integrated qualitative data to provide deeper explanatory insights into the mechanisms underlying FT. More critically, however, this difference stems from divergent healthcare financing systems between the United States and China. In the United States, financial burden is often influenced by insurance complexity, including high deductibles and co‐payments. In contrast, within China's comprehensive basic medical insurance framework, the primary contributor to FT among patients with NMOSD appears to be substantial out‐of‐pocket costs for high‐cost biologic therapies—many of which remain excluded from the National Reimbursement Drug List (NRDL).

This is strongly supported by our qualitative data in which patients frequently identified “prohibitive drug costs” as their primary concern. Furthermore, the profile of key sociodemographic risk factors differs between settings. While the US study emphasized racial disparities as a major social determinant, our analysis identifies employment disruption, particularly “complete cessation,” as a dominant and independent risk factor. This contrast illustrates how the structure of social protection system shapes the principal dimensions of health‐related financial hardship; in China, the limited occupational safeguards for individuals with rare chronic conditions may render loss of income a more immediate and severe financial crisis.

In this study, we found that family income is a contributing factor to FT in NMOSD patients. Studies among cancer patients have also demonstrated that individuals with low income or unemployment are particularly susceptible to FT. (Politi et al. 2021; Meernik et al. 2021; Obeng‐Gyasi et al. 2021; Panzone et al. 2022). Qualitative interviews revealed that high out‐of‐pocket costs for expensive biologic agents, limited reimbursement for medical care received outside the local region, and income loss due to illness‐related disability were key stressors. Moreover, the physical demands and potential psychological burden associated with long‐term medication and treatment may impair patients’ work capacity and productivity over time (Moon et al. 2024). Similarly, family caregivers often invest substantial time and effort in patient care, leading to reduced personal income (Warner et al. 2022). Collectively, these factors contribute to decreased household income and increased financial burden.

The social support buffering theory suggests that social support may act as a protective factor during periods of stress (Huang et al. 2023). By providing material, emotional, and informational resources, social support can alleviate negative emotions associated with FT incidents and thereby improve patients’ health outcomes (J. Zhang et al. 2023). However, no significant association between social support and FT among patients was observed in this patient population.

This may be attributed to the fact that during interviews, certain family caregivers reported that relatives expressed only verbal concern without providing genuine assistance. Additionally, some individual family caregivers indicated that although their friends and relatives were aware of the patient's condition, they were unable to offer substantial support due to personal limitations. Concurrently, some families achieved temporary relief through measures such as spousal employment, children withdrawing from school, borrowing money, or liquidation of assets. However, prolonged reliance on loans or asset sales is unsustainable and accelerates the depletion of social support networks. Limited support from the health system further compounds the issue. This may reflect the uneven distribution of critical medical resources in China, ranging from diagnostic tools to qualified neurologists (Delgado‐Garcia et al. 2022). Moreover, the exclusion of certain high cost biologic agents from health insurance coverage and the stringent criteria for major illness assistance underscore the inadequacies of the current healthcare system. Notably, fear of burdening others led patients to avoid caregivers (Husić et al. 2025). This self‐perceived burden (SPB) manifests differently across cultures: in Western societies, it threatens autonomy (Dempsey et al. 2012); in China, it conflicts with “face” and filial piety (Qin et al. 2025), amplifying reluctance to accept charity. International evidence suggests SPB expression varies culturally, but the underlying distress is universal (GarcíaSarreón et al. 2025). Thus, purely economic interventions are insufficient; culturally sensitive psychosocial support is needed.

The results of this study indicate that patient self‐efficacy is associated with FT. Specifically, higher levels of self‐efficacy are associated with higher COST‐PROM scores and lower levels of FT. Self‐efficacy is defined as an individual's belief in their ability to overcome challenges and perform behaviors necessary to achieve desired outcomes (Bandura 1997). In the context of health‐related behaviors, greater self‐efficacy is linked to improved disease management and better clinical outcomes (Chen et al. 2025). Elevated self‐efficacy is associated with favorable outcomes across various chronic disease populations, including reduced depressive symptoms (Cai et al. 2024, S. Zhang et al. 2022), enhanced self‐regulation (Giesler and Weis 2021), decreased symptom burden (Gao et al. 2022; Ying et al. 2025), and improved quality of life (Younas et al. 2024; Batool et al. 2024). During interviews, patients with higher self‐efficacy were more likely to employ positive coping strategies to manage financial difficulties arising illness. They mitigated both financial burden and psychological distress by actively seeking various forms of financial assistance and social support.

Higher fear of disease progression scores were associated with increased levels of FT, consistent with findings from cancer populations (Shen et al. 2022; Xiong et al. 2025). Negative emotions exacerbated FT in patients (Xiong et al. 2025). NMOSD patients were found to be prone to psychological stress and anxiety due to the chronic nature of the disease and its relapsing tendency, expressing concerns about disease progression and adverse outcomes and consequently experiencing varying degrees of fear.

At the same time, several interview findings warrant serious consideration. Behaviors such as opting for suboptimal treatments or avoiding policy‐related applications due to financial constraints in some patients, although reducing short‐term expenditures, ultimately lead to increased medical and social costs in the long term. This aligns with the concept of bounded rationality in health behavior theory (Simon 1955), which posits that patients tend to make locally rather than globally optimal decisions under conditions of information asymmetry and limited resouces. These cognitive biases further exacerbate patient FT.

The complex interplay of economic, clinical, and psychosocial factors identified in this study, along with the nuanced coping strategies reported by patients, indicates that FT in NMOSD is a multidimensional and likely heterogeneous phenomenon. Therefore, advancing beyond a binary “high/low” risk classification toward identifying distinct patient subgroups based on their specific profiles of financial distress, available resources, and coping mechanisms may represent a promising avenue for future research. Such person‐centered approaches could facilitate the development of more targeted and effective supportive interventions.

There is no doubt that the Chinese government has consistently demonstrated its commitment to supporting patients. Since 2009, China has established a comprehensive health insurance system through deepening healthcare reform and launched the “Healthy China 2030” initiative (Debie et al. 2022). To reduce disparities in per capita premiums and funding source, the government has implemented an outpatient catastrophic illness insurance scheme to enhance reimbursement rates and raise the annual reimbursement ceiling. Furthermore, a critical illness insurance system encompassing both urban and rural populations has been established, aiming to break the cycle of poverty by protecting low‐income groups from financial catastrophe due to high medical expenditures (Zhong et al. 2021; Tao et al. 2020). Nevertheless, patients continue to face substantial financial burdens stemming from unequal distribution of healthcare resources and rising medical costs. This study underscores the importance of proactively assessing FT in patients with NMOSD in clinical practice in order to interrupt the cascade of crises arising from household economic collapse, rapid deterioration in quality of life, and impaired psychosocial functioning.

Therefore, the following measures should be implemented to mitigate the FT on NMOSD patients: (1) The government should increase funding for health insurance coverage of rare diseases such as NMOSD, expand the scope of reimbursable medical services, include more effective therapeutic agents in the national medical insurance drug list, and raise reimbursement rates. Simultaneously, the reimbursement process should be streamlined to reduce the administrative procedures and time cost for patients. (2) Strengthen primary care standards and promote tiered diagnosis and treatment systems to alleviate shortage in primary healthcare resources. (3) Enhance public awareness and patient education, improve understanding of relevant policies, and collaborate with nonprofit organizations to reduce stigma. (4) Reinforce the social assistance framework and broaden channels for aid. The government can increase support for charitable initiatives and encourage nongovernmental organizations to establish dedicated relief funds for NMOSD patients experiencing financial hardship. (5) A robust social support system can help alleviate negative emotional outcomes such as depression and anxiety. Non‐professional caregivers can provide practical and material assistance, thereby reducing overall financial strain. It is also recommended to strengthen communication among neurologists, nurses, and patients. Discussions regarding healthcare costs are integral to maintaining quality of life; through transparent cost communication, patients can better understand their financial situation and access appropriate support from various sectors.

### Limitations

This study has several limitations that should be taken into account when interpreting the results. First, FT was measured using the COST‐PROM instrument, which is based on Classical Test Theory and has not been validated with contemporary psychometric methods—such as Rasch or item response theory analysis—within the NMOSD population. Although the instrument demonstrated acceptable internal consistency in our sample, this may limit the precision of its scores as an interval‐level measure. Second, the study utilized a cross‐sectional design at a single tertiary referral center. While this center serves a large patient population and our sample exhibited varied disease severity, the findings may not be fully generalizable to NMOSD patients in other regions of China, those treated in community‐based settings, or individuals who are undiagnosed or not engaged with the healthcare system. Third, although consecutive sampling of inpatients was employed to minimize selection bias, the sample was inherently restricted to individuals receiving regular hospital‐based care, potentially overrepresenting those with higher disease activity or better access to healthcare resources. Fourth, reliance on self‐reported data for clinical characteristics (e.g., relapse history) and socioeconomic factors may introduce recall bias and social desirability bias. Finally, although key confounders were adjusted for in our multivariate models, residual confounding by unmeasured variables—such as detailed asset‐to‐debt ratios, specific insurance plan co‐payment structures, or informal caregiving support—cannot be excluded, and the cross‐sectional design precludes causal inference regarding the observed associations. Nonetheless, we believe these findings contribute to the ongoing discussion on factors linked to FT in NMOSD patients. The results offer valuable insights for future research exploring determinants of FT in this population.

## Author Contributions

**Bo Wang**:study conception/design, data collection, analysis/interpretation, manuscript drafting. **Shuchuan Tian**: study conception/design and data collection. **Zhaoxia Wang**: Data collection. **Guilian Wang**: Data collection. **Li Li**: Data collection. **Jie Li**: Data collection. **Huaxing Meng**: Study conception/design, data collection, data interpretation, critical manuscript revision. All authors approved the final manuscript.

## Funding

This study was supported by Sanjin Elite Youth Top notch Talent Project fund (Grant No. SJYC2024439).

## Ethics Statement

Approval for this research endeavor was obtained from the Ethics Committee of the First Hospital under Shanxi Medical University, and each participant furnished written proofs to affirm their informed consent.

## Conflicts of Interest

The authors declare no Conflicts of Interest.

## Data Availability

Data are available from the corresponding author upon reasonable request.
